# *cis*-Aqua­bis(2,2′-bipyridine-κ^2^ *N*,*N*′)fluoridochromium(III) bis­(perchlorate) dihydrate

**DOI:** 10.1107/S1600536810000127

**Published:** 2010-01-09

**Authors:** Torben Birk, Jesper Bendix

**Affiliations:** a Department of Chemistry, University of Copenhagen, Universitetsparken 5, DK.2100 Copenhagen Ø, Denmark

## Abstract

The title mixed aqua–fluoride complex, [CrF(C_10_H_8_N_2_)_2_(H_2_O)](ClO_4_)_2_·2H_2_O, has been synthesized by aqua­tion of the corresponding difluoride complex using lanthan­ide(III) ions as F^−^ acceptors. The complex crystallizes with a Cr^III^ ion at the center of a distorted octa­hedral coordination polyhedron with a *cis* arrangement of ligands. The crystal packing shows a hydrogen-bonding pattern involving water mol­ecules, the coordinated F atom and the perchlorate anions

## Related literature

For related difluoride complexes, see: Birk *et al.* (2008 ▶); Brenčič *et al.* (1987 ▶); Brenčič & Leban (1981 ▶); DeJovine *et al.* (1974 ▶); Delavar & Staples (1981 ▶); Kavitha *et al.* (2005 ▶); Vaughn *et al.* (1968 ▶); Vaughn & Seiler (1979 ▶); Yamaguchi-Terasaki *et al.* (2007 ▶). For related structures, see: Casellato *et al.* (1986 ▶); Liu (2009 ▶). For details of the synthesis, see: Glerup *et al.* (1970 ▶).
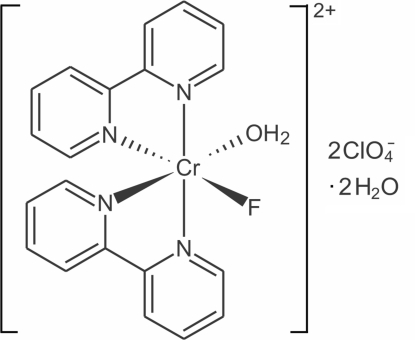


## Experimental

### 

#### Crystal data

[CrF(C_10_H_8_N_2_)_2_(H_2_O)](ClO_4_)_2_·2H_2_O
*M* *_r_* = 636.32
Triclinic, 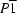

*a* = 9.577 (1) Å
*b* = 11.4050 (6) Å
*c* = 11.8150 (11) Å
α = 77.273 (6)°
β = 79.427 (9)°
γ = 83.590 (5)°
*V* = 1234.01 (19) Å^3^
*Z* = 2
Mo *K*α radiation
μ = 0.76 mm^−1^
*T* = 122 K
0.41 × 0.24 × 0.14 mm

#### Data collection

Nonius KappaCCD diffractometer
Absorption correction: Gaussian (Coppens, 1970 ▶) *T*_min_ = 0.805, *T*_max_ = 0.925
27824 measured reflections
5691 independent reflections
5244 reflections with *I* > 2σ(*I*)
*R*_int_ = 0.021

#### Refinement

*R*[*F* ^2^ > 2σ(*F* ^2^)] = 0.026
*wR*(*F* ^2^) = 0.070
*S* = 1.03
5691 reflections
352 parameters
H-atom parameters constrained
Δρ_max_ = 0.55 e Å^−3^
Δρ_min_ = −0.42 e Å^−3^

### 

Data collection: *COLLECT* (Nonius, 1998 ▶); cell refinement: *COLLECT*; data reduction: *EVALCCD* (Duisenberg *et al.*, 2003 ▶); program(s) used to solve structure: *SHELXS97* (Sheldrick, 2008 ▶); program(s) used to refine structure: *SHELXL97* (Sheldrick, 2008 ▶); molecular graphics: *ORTEP-3* (Farrugia, 1997 ▶); software used to prepare material for publication: *SHELXL97*.

## Supplementary Material

Crystal structure: contains datablocks global, I. DOI: 10.1107/S1600536810000127/hy2268sup1.cif

Structure factors: contains datablocks I. DOI: 10.1107/S1600536810000127/hy2268Isup2.hkl

Additional supplementary materials:  crystallographic information; 3D view; checkCIF report

## Figures and Tables

**Table 1:** Selected bond lengths (Å)

Cr1—F1	1.8614 (8)
Cr1—O1	1.9579 (10)
Cr1—N1	2.0501 (12)
Cr1—N2	2.0456 (12)
Cr1—N3	2.0545 (12)
Cr1—N4	2.0571 (12)

**Table 2:** Hydrogen-bond geometry (Å, °)

*D*—H⋯*A*	*D*—H	H⋯*A*	*D*⋯*A*	*D*—H⋯*A*
O1—H1*A*⋯F1^i^	0.83	1.73	2.5482 (13)	174
O1—H1*B*⋯O2^ii^	0.83	1.73	2.5548 (15)	176
O2—H2*A*⋯O3	0.90	1.89	2.7887 (18)	179
O2—H2*B*⋯O5	0.84	2.14	2.9380 (17)	158
O3—H3*A*⋯O10^iii^	0.91	2.00	2.890 (2)	167
O3—H3*B*⋯O8	0.87	2.19	3.050 (2)	168
O3—H3*B*⋯O9	0.87	2.48	3.123 (2)	132

Symmetry codes: (i) 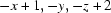; (ii) 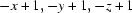; (iii) 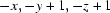.

**Table 3:** *M*—F bond distances (Å) for related *cis/*trans**-[*M*(*L*)_2_F_2_]^+^ complexes

(I)	(II)	(III)	(IV)	(V)
1.8621 (10)	1.8541 (10)	1.887 (6)	1.887 (5)	1.7389 (15)
1.8444 (10)	1.8409 (10)	1.878 (6)	1.868 (4)	1.7232 (15)

Notes: (I) *cis*-[Cr(phen)_2_F_2_]ClO_4_·H_2_O (Birk *et al.*, 2008 ▶); (II) *cis*-[Cr(bipy)_2_F_2_]ClO_4_·H_2_O (Yamaguchi-Terasaki *et al.*, 2007 ▶); (III) *trans*-[Cr(en)_2_F_2_]ClO_4_ (Brenčič & Leban, 1981 ▶); (IV) *cis*-[Cr(en)_2_F_2_]ClO_4_·NaClO_4_·H_2_O (Brenčič *et al.*, 1987 ▶); (V) *cis*-[V(bipy)_2_F_2_]BF_4_ (Kavitha *et al.*, 2005 ▶). en = ethane-1,2-diamine; bipy = 2,2′-bipyridine; phen = 1,10-phenanthroline.

**Fig. 1.**
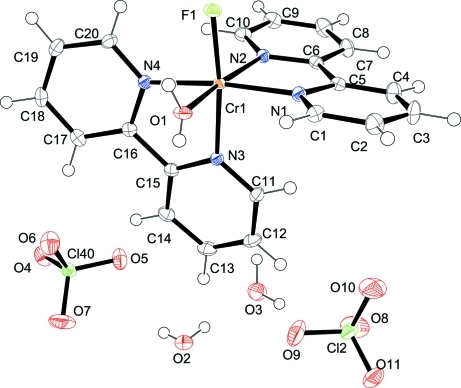
The molecular structure of the title compound. Displacement ellipsoids are drawn at the 50% probability.

**Fig. 2.**
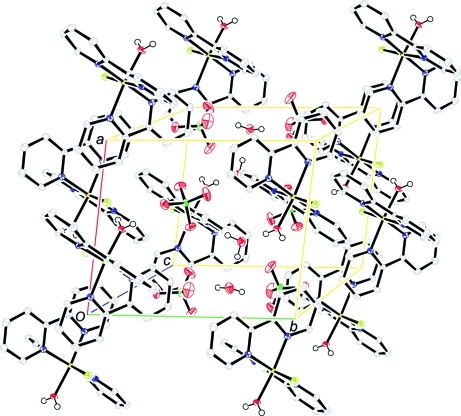
The crystal packing in the title compound. Displacement ellipsoids are drawn at the 50% probability. H atoms except those of water molecules have been omitted for clarity.

## supplementary crystallographic information

### Comment

Water and fluoride are both hard donor ligands favoring the same central ions. Nevertheless, no transition metal complexes of the general type *cis*-[M(*L*)_2_(H_2_O)F]^2+^ with *L* being a bidentate ligand such as 2,2'-bipyridine (bipy), 1,10-phenanthroline (phen) or ethane-1,2-diamine (en) have previously been characterized by X-ray diffraction. This report presents the synthesis and crystal structure of such a system exemplified by the title complex *cis*-[Cr(bipy)_2_(H_2_O)F](ClO_4_)_2_.2H_2_O.

All trivalent lanthanid ions, Ln^3+^ are known to be hard Lewis acids. This Lewis acidity gives rise to favorable bond formation with ligands containing oxygen and fluorine ligator atoms. The interactions between lanthanid ions and coordinated fluoride have not received much attention compared to the plethora of oxygen bridged systems reported. We have initiated a study on the reactivity of lanthanid ions towards coordinated fluoride ligands to assess if new fluoride contaning complexes can be synthesized this way. In the context of these studies, we found that the title complex, as well as the corresponding phen complex, can be synthesized by a lanthanid ion assisted aquation of the difluoride complex *cis*-[Cr(bipy)_2_F_2_]ClO_4_. The normal aquation reaction is performed in strong acid and has been studied preparatively and kinetic for difluoride complexes as *trans*-[Cr(en)_2_F_2_]^+^ and *cis*-[Cr(bipy)_2_F_2_]^+^ (DeJovine *et al.*, 1974; Delavar & Staples, 1981; Vaughn *et al.*, 1968; Vaughn & Seiler, 1979). However, the present synthetic approach is more general, as it can be applied also to systems with an acid-labile auxillary ligand sphere.

The most important structural element in the title compound is the *cis* arrangement of ligators in a distorted octahedral coordination polyhedron around the central Cr^III^ ion (Fig. 1). Distortion from ideal geometry is dictated by the nearly fixed bite angles of the two bipy ligands [79.51 (5) and 79.24 (5)°]. This pattern is also seen in the structurally related difluoride complexes *cis*-[V(bipy)_2_F_2_]BF_4_ (Kavitha *et al.*, 2005) and *cis*-[Cr(bipy)F_2_]ClO_4_.H_2_O (Yamaguchi-Terasaki *et al.*, 2007). The Cr1—F1 bond distance of 1.8614 (8) Å (Table 1) is in accordance with the structurally characterized difluoride complexes, as shown in Table 3. The Cr1—O1 bond distance of 1.9579 (10)Å is shorter than what is seen in both the tricationic, diaqua complex *cis*-[Cr(bipy)_2_(H_2_O)_2_](NO_3_)_3_ [2.00 (1) and 1.98 (1) Å] (Casellato *et al.*, 1986), as well as in the uncharged [Cr(bipy)(H_2_O)F_3_].2H_2_O [1.979 (2) Å] (Liu, 2009). In the structure of *cis*-[Cr(bipy)_2_F_2_]^+^, a *trans* influence leading to the Cr—N bond distances *trans* to the fluorido ligand being longer that the corresponding *cis* distances was identified by Yamaguchi-Terasaki *et al.* (2007). This situation was also found in *cis*-[Cr(phen)_2_F_2_]^+^ (Birk *et al.*, 2008), but is not discernible in the structure reported here.

The crystal packing in the title complex (Fig. 2) shows a hydrogen bonding pattern involving water molecules, coordinated F atom and perchlorate anions (Table 2).

### Experimental

*Safety remark:* Perchlorate complexes of metal ions are potentially explosive. The title complex burns with high intensity when ignited in a gas flame. According to Delavar & Staples (1981), the corresponding phen complex is explosive.

All chemicals were used as received. *cis*-[Cr(bipy)_2_F_2_]ClO_4_ was synthesized by reflux of *trans*-difluorotetrakis(pyridine)chromium(III) perchlorate and bipy in 2-methoxyethanol according to the published method (Glerup *et al.*, 1970).

Nd_2_O_3_ (0.251 g, 0.746 mmol) was dissolved in 0.5 ml HClO_4_ (60%) by gentle heating giving a pink solution to which was added a mixture of *cis*-[Cr(bipy)_2_F_2_]ClO_4_ (1.011 g, 2.015 mmol) in acteonitrile (60 ml) and water (20 ml). Mixing gave rise to a slight color change from purple to red. The solution was placed in a water bath (~70°C) and was stirred for 30–35 min. The resulting muddy red suspension was solidified by cooling to room temperature. Extraction with a mixture of acetonitrile and water (2:1, 30 ml) gave an orange turbid solution which was separated into a clear solution and white precipitate by centrifugation. Slow evaporation gave an orange crystalline product which contained crystals of suitable quality for X-ray diffraction. The product was isolated by filtering, washed with ice water and dried in air (yield 1.073 g, 83.7%). Analysis, calculated for C_20_H_22_Cl_2_CrFN_4_O_11_: C 37.75, H 3.48, N 8.80%; found: C 37.97, H 3.41, N 8.70%.

The corresponding phen complex, *cis*-[Cr(phen)_2_(H_2_O)F](ClO_4_)_2_.H_2_O can be synthesized from *cis*-[Cr(phen)_2_F_2_]ClO_4_ (1.034 g, 1.88 mmol) by a similar procedure (yield 0.781 g, 60.7%). Analysis, calculated for C_24_H_22_Cl_2_CrFN_4_O_11_: C 42.12, H 3.24, N 8.19%; found: C 42.07, H 2.73, N 8.02%.

### Refinement

The aromatic H atoms were placed in geometrically idealized positions and refined as riding atoms, with C—H = 0.95 Å and with *U*_iso_(H) = 1.2*U*_eq_(C). H atoms of water molecules were identified in a difference Fourier map and refined as riding with *U*_iso_(H) = 1.2*U*_eq_(O).

### Crystal data

**Table:**

[CrF(C_10_H_8_N_2_)_2_(H_2_O)](ClO_4_)_2_·2H_2_O	*Z* = 2
*M**_r_* = 636.32	*F*(000) = 650
Triclinic, *P*1	*D*_x_ = 1.712 Mg m^−^^3^
Hall symbol: -P 1	Mo *K*α radiation, λ = 0.71073 Å
*a* = 9.577 (1) Å	Cell parameters from 19362 reflections
*b* = 11.4050 (6) Å	θ = 1.8–27.6°
*c* = 11.8150 (11) Å	µ = 0.76 mm^−^^1^
α = 77.273 (6)°	*T* = 122 K
β = 79.427 (9)°	Block, orange
γ = 83.590 (5)°	0.41 × 0.24 × 0.14 mm
*V* = 1234.01 (19) Å^3^	

### Data collection

**Table:**

Nonius KappaCCD diffractometer	5691 independent reflections
Radiation source: fine-focus sealed tube	5244 reflections with *I* > 2σ(*I*)
graphite	*R*_int_ = 0.021
ω and φ scans	θ_max_ = 27.6°, θ_min_ = 1.8°
Absorption correction: gaussian (Coppens, 1970)	*h* = −12→12
*T*_min_ = 0.805, *T*_max_ = 0.925	*k* = −14→11
27824 measured reflections	*l* = −15→15

### Refinement

**Table:**

Refinement on *F*^2^	Primary atom site location: structure-invariant direct methods
Least-squares matrix: full	Secondary atom site location: difference Fourier map
*R*[*F*^2^ > 2σ(*F*^2^)] = 0.026	Hydrogen site location: inferred from neighbouring sites
*wR*(*F*^2^) = 0.070	H-atom parameters constrained
*S* = 1.03	*w* = 1/[σ^2^(*F*_o_^2^) + (0.0294*P*)^2^ + 1.0724*P*] where *P* = (*F*_o_^2^ + 2*F*_c_^2^)/3
5691 reflections	(Δ/σ)_max_ = 0.001
352 parameters	Δρ_max_ = 0.55 e Å^−^^3^
0 restraints	Δρ_min_ = −0.42 e Å^−^^3^

### Fractional atomic coordinates and isotropic or equivalent isotropic displacement parameters (Å^2^)

**Table:**

	*x*	*y*	*z*	*U*_iso_*/*U*_eq_	
Cr1	0.35121 (2)	0.123062 (19)	0.848099 (18)	0.00991 (6)	
Cl2	0.01627 (4)	0.74580 (3)	0.50375 (3)	0.02144 (9)	
N3	0.35227 (12)	0.26595 (10)	0.70747 (10)	0.0118 (2)	
N1	0.22015 (12)	0.21617 (10)	0.96126 (10)	0.0126 (2)	
N2	0.15621 (12)	0.08156 (10)	0.82758 (10)	0.0126 (2)	
N4	0.47511 (12)	0.04749 (10)	0.71710 (10)	0.0127 (2)	
O9	0.15767 (16)	0.70270 (13)	0.45676 (13)	0.0404 (3)	
C11	0.27881 (15)	0.37340 (13)	0.70730 (13)	0.0157 (3)	
H11	0.2249	0.3889	0.7791	0.019*	
C10	0.13318 (16)	0.00256 (13)	0.76532 (12)	0.0166 (3)	
H10	0.2127	−0.0408	0.7285	0.020*	
C5	0.07893 (15)	0.21767 (12)	0.95836 (12)	0.0138 (3)	
C6	0.04290 (15)	0.14086 (12)	0.88386 (12)	0.0136 (3)	
C16	0.51342 (15)	0.12642 (12)	0.61489 (12)	0.0134 (3)	
O8	−0.07727 (16)	0.74614 (14)	0.42147 (13)	0.0422 (4)	
C12	0.27874 (16)	0.46239 (13)	0.60644 (14)	0.0191 (3)	
H12	0.2266	0.5379	0.6092	0.023*	
C1	0.26327 (16)	0.27937 (13)	1.03060 (12)	0.0164 (3)	
H1	0.3622	0.2785	1.0322	0.020*	
C13	0.35579 (17)	0.43951 (14)	0.50168 (13)	0.0201 (3)	
H13	0.3546	0.4981	0.4308	0.024*	
C20	0.54167 (15)	−0.06375 (13)	0.73489 (13)	0.0162 (3)	
H20	0.5130	−0.1192	0.8061	0.019*	
C4	−0.02135 (16)	0.28464 (15)	1.02335 (14)	0.0215 (3)	
H4	−0.1196	0.2867	1.0187	0.026*	
O11	0.02074 (14)	0.86579 (11)	0.52039 (12)	0.0325 (3)	
C2	0.16777 (17)	0.34562 (14)	1.09959 (14)	0.0213 (3)	
H2	0.2005	0.3883	1.1490	0.026*	
C14	0.43510 (16)	0.32978 (14)	0.50111 (13)	0.0183 (3)	
H14	0.4903	0.3131	0.4302	0.022*	
C18	0.69355 (16)	−0.01741 (14)	0.54925 (14)	0.0203 (3)	
H18	0.7703	−0.0392	0.4925	0.024*	
C9	−0.00262 (17)	−0.01809 (14)	0.75276 (13)	0.0205 (3)	
H9	−0.0162	−0.0742	0.7078	0.025*	
C15	0.43247 (14)	0.24527 (12)	0.60527 (12)	0.0132 (3)	
C8	−0.11776 (17)	0.04497 (15)	0.80722 (14)	0.0229 (3)	
H8	−0.2118	0.0340	0.7984	0.028*	
C7	−0.09549 (16)	0.12434 (14)	0.87471 (13)	0.0195 (3)	
H7	−0.1739	0.1667	0.9140	0.023*	
O10	−0.03306 (19)	0.66919 (14)	0.61505 (13)	0.0487 (4)	
C17	0.62360 (16)	0.09676 (14)	0.52947 (13)	0.0180 (3)	
H17	0.6506	0.1535	0.4588	0.022*	
C3	0.02397 (17)	0.34882 (15)	1.09551 (15)	0.0247 (3)	
H3	−0.0432	0.3944	1.1416	0.030*	
C19	0.65089 (16)	−0.09956 (13)	0.65221 (14)	0.0191 (3)	
H19	0.6957	−0.1789	0.6658	0.023*	
Cl40	0.60695 (4)	0.35453 (3)	0.15860 (3)	0.01724 (8)	
O4	0.61978 (13)	0.29094 (12)	0.06429 (11)	0.0284 (3)	
O5	0.45771 (12)	0.37385 (12)	0.20582 (11)	0.0296 (3)	
O6	0.67943 (14)	0.28330 (12)	0.25003 (11)	0.0322 (3)	
O7	0.66883 (17)	0.46781 (12)	0.11571 (13)	0.0397 (3)	
F1	0.36386 (9)	−0.01840 (7)	0.96103 (7)	0.01657 (17)	
O2	0.37076 (13)	0.62663 (10)	0.21825 (10)	0.0241 (2)	
H2A	0.2792	0.6097	0.2348	0.029*	
H2B	0.4116	0.5629	0.1987	0.029*	
O3	0.08500 (14)	0.57629 (12)	0.26794 (11)	0.0298 (3)	
H3A	0.0722	0.5022	0.3148	0.036*	
H3B	0.0491	0.6222	0.3174	0.036*	
O1	0.52507 (10)	0.17280 (9)	0.88317 (9)	0.0153 (2)	
H1A	0.5620	0.1268	0.9357	0.018*	
H1B	0.5629	0.2360	0.8503	0.018*	

### Atomic displacement parameters (Å^2^)

**Table:**

	*U*^11^	*U*^22^	*U*^33^	*U*^12^	*U*^13^	*U*^23^
Cr1	0.00962 (10)	0.01015 (11)	0.00923 (10)	−0.00046 (7)	−0.00176 (7)	−0.00038 (8)
Cl2	0.0299 (2)	0.01514 (16)	0.01916 (17)	0.00224 (13)	−0.00580 (14)	−0.00367 (13)
N3	0.0114 (5)	0.0123 (5)	0.0118 (5)	−0.0013 (4)	−0.0029 (4)	−0.0014 (4)
N1	0.0133 (5)	0.0134 (5)	0.0104 (5)	−0.0010 (4)	−0.0017 (4)	−0.0013 (4)
N2	0.0130 (5)	0.0134 (5)	0.0106 (5)	−0.0018 (4)	−0.0022 (4)	−0.0003 (4)
N4	0.0117 (5)	0.0124 (5)	0.0137 (5)	−0.0012 (4)	−0.0017 (4)	−0.0024 (4)
O9	0.0434 (8)	0.0351 (7)	0.0369 (7)	0.0189 (6)	−0.0020 (6)	−0.0098 (6)
C11	0.0157 (6)	0.0138 (6)	0.0169 (7)	0.0003 (5)	−0.0027 (5)	−0.0024 (5)
C10	0.0188 (7)	0.0169 (7)	0.0143 (6)	−0.0024 (5)	−0.0019 (5)	−0.0037 (5)
C5	0.0136 (6)	0.0150 (6)	0.0122 (6)	−0.0015 (5)	−0.0020 (5)	−0.0010 (5)
C6	0.0135 (6)	0.0147 (6)	0.0116 (6)	−0.0014 (5)	−0.0019 (5)	−0.0005 (5)
C16	0.0132 (6)	0.0142 (6)	0.0135 (6)	−0.0024 (5)	−0.0031 (5)	−0.0030 (5)
O8	0.0470 (8)	0.0426 (8)	0.0440 (8)	−0.0172 (7)	−0.0238 (7)	−0.0032 (6)
C12	0.0193 (7)	0.0132 (6)	0.0235 (7)	0.0005 (5)	−0.0063 (6)	0.0007 (6)
C1	0.0174 (7)	0.0180 (7)	0.0145 (6)	−0.0028 (5)	−0.0035 (5)	−0.0034 (5)
C13	0.0237 (7)	0.0173 (7)	0.0173 (7)	−0.0033 (6)	−0.0069 (6)	0.0047 (5)
C20	0.0167 (7)	0.0129 (6)	0.0187 (7)	−0.0010 (5)	−0.0034 (5)	−0.0025 (5)
C4	0.0147 (7)	0.0274 (8)	0.0234 (8)	0.0011 (6)	−0.0016 (6)	−0.0101 (6)
O11	0.0370 (7)	0.0230 (6)	0.0415 (7)	0.0011 (5)	−0.0069 (6)	−0.0164 (5)
C2	0.0241 (8)	0.0235 (7)	0.0192 (7)	−0.0025 (6)	−0.0030 (6)	−0.0108 (6)
C14	0.0212 (7)	0.0194 (7)	0.0131 (7)	−0.0034 (6)	−0.0017 (5)	−0.0004 (5)
C18	0.0157 (7)	0.0249 (8)	0.0206 (7)	0.0010 (6)	0.0002 (6)	−0.0093 (6)
C9	0.0234 (8)	0.0226 (7)	0.0182 (7)	−0.0079 (6)	−0.0048 (6)	−0.0058 (6)
C15	0.0129 (6)	0.0139 (6)	0.0131 (6)	−0.0028 (5)	−0.0023 (5)	−0.0020 (5)
C8	0.0164 (7)	0.0304 (8)	0.0242 (8)	−0.0076 (6)	−0.0048 (6)	−0.0062 (6)
C7	0.0132 (7)	0.0251 (7)	0.0205 (7)	−0.0025 (6)	−0.0015 (5)	−0.0058 (6)
O10	0.0745 (11)	0.0352 (8)	0.0266 (7)	−0.0070 (7)	0.0050 (7)	0.0049 (6)
C17	0.0164 (7)	0.0208 (7)	0.0156 (7)	−0.0025 (5)	0.0006 (5)	−0.0033 (5)
C3	0.0224 (8)	0.0286 (8)	0.0247 (8)	0.0030 (6)	0.0003 (6)	−0.0149 (7)
C19	0.0175 (7)	0.0161 (7)	0.0244 (8)	0.0031 (5)	−0.0040 (6)	−0.0074 (6)
Cl40	0.01915 (17)	0.01732 (16)	0.01546 (16)	−0.00394 (12)	−0.00042 (12)	−0.00447 (12)
O4	0.0287 (6)	0.0368 (7)	0.0245 (6)	−0.0081 (5)	0.0006 (5)	−0.0180 (5)
O5	0.0193 (6)	0.0376 (7)	0.0300 (6)	0.0045 (5)	0.0003 (5)	−0.0095 (5)
O6	0.0287 (6)	0.0422 (7)	0.0226 (6)	0.0053 (5)	−0.0068 (5)	−0.0023 (5)
O7	0.0553 (9)	0.0245 (6)	0.0397 (8)	−0.0214 (6)	−0.0010 (7)	−0.0043 (6)
F1	0.0164 (4)	0.0153 (4)	0.0157 (4)	−0.0020 (3)	−0.0043 (3)	0.0034 (3)
O2	0.0263 (6)	0.0159 (5)	0.0277 (6)	−0.0039 (4)	−0.0016 (5)	−0.0003 (4)
O3	0.0345 (7)	0.0294 (6)	0.0262 (6)	−0.0073 (5)	0.0015 (5)	−0.0100 (5)
O1	0.0143 (5)	0.0140 (5)	0.0170 (5)	−0.0037 (4)	−0.0067 (4)	0.0031 (4)

### Geometric parameters (Å, °)

**Table:**

Cr1—F1	1.8614 (8)	C13—C14	1.390 (2)
Cr1—O1	1.9579 (10)	C13—H13	0.9500
Cr1—N1	2.0501 (12)	C20—C19	1.386 (2)
Cr1—N2	2.0456 (12)	C20—H20	0.9500
Cr1—N3	2.0545 (12)	C4—C3	1.390 (2)
Cr1—N4	2.0571 (12)	C4—H4	0.9500
Cl2—O11	1.4311 (12)	C2—C3	1.383 (2)
Cl2—O8	1.4369 (14)	C2—H2	0.9500
Cl2—O10	1.4400 (14)	C14—C15	1.3841 (19)
Cl2—O9	1.4421 (14)	C14—H14	0.9500
N3—C11	1.3431 (18)	C18—C19	1.388 (2)
N3—C15	1.3595 (18)	C18—C17	1.388 (2)
N1—C1	1.3460 (18)	C18—H18	0.9500
N1—C5	1.3573 (18)	C9—C8	1.383 (2)
N2—C10	1.3408 (18)	C9—H9	0.9500
N2—C6	1.3589 (18)	C8—C7	1.387 (2)
N4—C20	1.3458 (18)	C8—H8	0.9500
N4—C16	1.3548 (18)	C7—H7	0.9500
C11—C12	1.385 (2)	C17—H17	0.9500
C11—H11	0.9500	C3—H3	0.9500
C10—C9	1.387 (2)	C19—H19	0.9500
C10—H10	0.9500	Cl40—O7	1.4316 (13)
C5—C4	1.385 (2)	Cl40—O4	1.4382 (12)
C5—C6	1.4764 (19)	Cl40—O6	1.4392 (12)
C6—C7	1.386 (2)	Cl40—O5	1.4470 (12)
C16—C17	1.388 (2)	O2—H2A	0.8959
C16—C15	1.4769 (19)	O2—H2B	0.8435
C12—C13	1.381 (2)	O3—H3A	0.9096
C12—H12	0.9500	O3—H3B	0.8684
C1—C2	1.384 (2)	O1—H1A	0.8255
C1—H1	0.9500	O1—H1B	0.8276
			
F1—Cr1—O1	90.26 (4)	N1—C1—H1	119.0
F1—Cr1—N2	89.57 (4)	C2—C1—H1	119.0
O1—Cr1—N2	172.88 (5)	C12—C13—C14	119.24 (13)
F1—Cr1—N1	94.23 (4)	C12—C13—H13	120.4
O1—Cr1—N1	93.41 (5)	C14—C13—H13	120.4
N2—Cr1—N1	79.51 (5)	N4—C20—C19	121.80 (13)
F1—Cr1—N3	172.26 (4)	N4—C20—H20	119.1
O1—Cr1—N3	90.13 (4)	C19—C20—H20	119.1
N2—Cr1—N3	90.99 (5)	C5—C4—C3	119.00 (14)
N1—Cr1—N3	93.46 (5)	C5—C4—H4	120.5
F1—Cr1—N4	93.04 (4)	C3—C4—H4	120.5
O1—Cr1—N4	88.50 (5)	C3—C2—C1	119.02 (14)
N2—Cr1—N4	98.62 (5)	C3—C2—H2	120.5
N1—Cr1—N4	172.47 (5)	C1—C2—H2	120.5
N3—Cr1—N4	79.24 (5)	C15—C14—C13	119.05 (14)
O11—Cl2—O8	109.72 (9)	C15—C14—H14	120.5
O11—Cl2—O10	109.32 (9)	C13—C14—H14	120.5
O8—Cl2—O10	110.21 (10)	C19—C18—C17	119.71 (14)
O11—Cl2—O9	108.75 (9)	C19—C18—H18	120.1
O8—Cl2—O9	108.89 (9)	C17—C18—H18	120.1
O10—Cl2—O9	109.91 (9)	C8—C9—C10	118.43 (14)
C11—N3—C15	118.59 (12)	C8—C9—H9	120.8
C11—N3—Cr1	126.26 (10)	C10—C9—H9	120.8
C15—N3—Cr1	115.12 (9)	N3—C15—C14	121.69 (13)
C1—N1—C5	119.17 (12)	N3—C15—C16	114.74 (12)
C1—N1—Cr1	125.61 (10)	C14—C15—C16	123.57 (13)
C5—N1—Cr1	115.10 (9)	C9—C8—C7	119.79 (14)
C10—N2—C6	119.20 (12)	C9—C8—H8	120.1
C10—N2—Cr1	125.76 (10)	C7—C8—H8	120.1
C6—N2—Cr1	115.04 (9)	C6—C7—C8	118.95 (14)
C20—N4—C16	119.50 (12)	C6—C7—H7	120.5
C20—N4—Cr1	124.11 (10)	C8—C7—H7	120.5
C16—N4—Cr1	114.77 (9)	C16—C17—C18	118.73 (14)
N3—C11—C12	122.47 (13)	C16—C17—H17	120.6
N3—C11—H11	118.8	C18—C17—H17	120.6
C12—C11—H11	118.8	C2—C3—C4	119.37 (14)
N2—C10—C9	122.31 (14)	C2—C3—H3	120.3
N2—C10—H10	118.8	C4—C3—H3	120.3
C9—C10—H10	118.8	C20—C19—C18	118.75 (14)
N1—C5—C4	121.45 (13)	C20—C19—H19	120.6
N1—C5—C6	114.70 (12)	C18—C19—H19	120.6
C4—C5—C6	123.83 (13)	O7—Cl40—O4	109.83 (8)
N2—C6—C7	121.27 (13)	O7—Cl40—O6	109.51 (9)
N2—C6—C5	115.05 (12)	O4—Cl40—O6	109.40 (8)
C7—C6—C5	123.61 (13)	O7—Cl40—O5	110.01 (9)
N4—C16—C17	121.47 (13)	O4—Cl40—O5	108.98 (8)
N4—C16—C15	114.56 (12)	O6—Cl40—O5	109.10 (8)
C17—C16—C15	123.93 (13)	H2A—O2—H2B	101.9
C13—C12—C11	118.88 (14)	H3A—O3—H3B	100.6
C13—C12—H12	120.6	Cr1—O1—H1A	116.4
C11—C12—H12	120.6	Cr1—O1—H1B	125.2
N1—C1—C2	121.96 (14)	H1A—O1—H1B	118.3

### Hydrogen-bond geometry (Å, °)

**Table:**

*D*—H···*A*	*D*—H	H···*A*	*D*···*A*	*D*—H···*A*
O1—H1A···F1^i^	0.83	1.73	2.5482 (13)	174
O1—H1B···O2^ii^	0.83	1.73	2.5548 (15)	176
O2—H2A···O3	0.90	1.89	2.7887 (18)	179
O2—H2B···O5	0.84	2.14	2.9380 (17)	158
O3—H3A···O10^iii^	0.91	2.00	2.890 (2)	167
O3—H3B···O8	0.87	2.19	3.050 (2)	168
O3—H3B···O9	0.87	2.48	3.123 (2)	132

Symmetry codes: (i) −*x*+1, −*y*, −*z*+2; (ii) −*x*+1, −*y*+1, −*z*+1; (iii) −*x*, −*y*+1, −*z*+1.

### Table 3 *M*—F bond distances (Å) for related *cis/trans*-[*M*(*L*)_2_F_2_]^+^ complexes

**Table:**

(I)	(II)	(III)	(IV)	(V)
1.8621 (10)	1.8541 (10)	1.887 (6)	1.887 (5)	1.7389 (15)
1.8444 (10)	1.8409 (10)	1.878 (6)	1.868 (4)	1.7232 (15)

Notes: (I) *cis*-[Cr(phen)_2_F_2_]ClO_4_.H_2_O (Birk *et al.*, 2008); (II) *cis*-[Cr(bipy)_2_F_2_]ClO_4_.H_2_O (Yamaguchi-Terasaki *et al.*, 2007); (III) *trans*-[Cr(en)_2_F_2_]ClO_4_ (Brenčič & Leban, 1981); (IV) *cis*-[Cr(en)_2_F_2_]ClO_4_.NaClO_4_.H_2_O (Brenčič *et al.*, 1987); (V) *cis*-[V(bipy)_2_F_2_]BF_4_ (Kavitha *et al.*, 2005). en = ethane-1,2-diamine; bipy = 2,2'-bipyridine; phen = 1,10-phenanthroline.
